# Rational and Evolutionary Engineering Approaches Uncover a Small Set of Genetic Changes Efficient for Rapid Xylose Fermentation in *Saccharomyces cerevisiae*

**DOI:** 10.1371/journal.pone.0057048

**Published:** 2013-02-26

**Authors:** Soo Rin Kim, Jeffrey M. Skerker, Wei Kang, Anastashia Lesmana, Na Wei, Adam P. Arkin, Yong-Su Jin

**Affiliations:** 1 Department of Food Science and Human Nutrition, University of Illinois at Urbana-Champaign, Urbana, Illinois, United States of America; 2 Institute for Genomic Biology, University of Illinois at Urbana-Champaign, Urbana, Illinois, United States of America; 3 Department of Bioengineering, University of California, Berkeley, California, United States of America; 4 Physical Biosciences Division, Lawrence Berkeley National Laboratory, Berkeley, California, United States of America; Virginia Commonwealth University, United States of America

## Abstract

Economic bioconversion of plant cell wall hydrolysates into fuels and chemicals has been hampered mainly due to the inability of microorganisms to efficiently co-ferment pentose and hexose sugars, especially glucose and xylose, which are the most abundant sugars in cellulosic hydrolysates. *Saccharomyces cerevisiae* cannot metabolize xylose due to a lack of xylose-metabolizing enzymes. We developed a rapid and efficient xylose-fermenting *S. cerevisiae* through rational and inverse metabolic engineering strategies, comprising the optimization of a heterologous xylose-assimilating pathway and evolutionary engineering. Strong and balanced expression levels of the *XYL1*, *XYL2*, and *XYL3* genes constituting the xylose-assimilating pathway increased ethanol yields and the xylose consumption rates from a mixture of glucose and xylose with little xylitol accumulation. The engineered strain, however, still exhibited a long lag time when metabolizing xylose above 10 g/l as a sole carbon source, defined here as xylose toxicity. Through serial-subcultures on xylose, we isolated evolved strains which exhibited a shorter lag time and improved xylose-fermenting capabilities than the parental strain. Genome sequencing of the evolved strains revealed that mutations in *PHO13* causing loss of the Pho13p function are associated with the improved phenotypes of the evolved strains. Crude extracts of a *PHO13*-overexpressing strain showed a higher phosphatase activity on xylulose-5-phosphate (X-5-P), suggesting that the dephosphorylation of X-5-P by Pho13p might generate a futile cycle with xylulokinase overexpression. While xylose consumption rates by the evolved strains improved substantially as compared to the parental strain, xylose metabolism was interrupted by accumulated acetate. Deletion of *ALD6* coding for acetaldehyde dehydrogenase not only prevented acetate accumulation, but also enabled complete and efficient fermentation of xylose as well as a mixture of glucose and xylose by the evolved strain. These findings provide direct guidance for developing industrial strains to produce cellulosic fuels and chemicals.

## Introduction

Global climate changes and the soaring price of oil have evoked the desire for renewable and sustainable fuel production. A practical and sustainable solution to the energy crisis is to produce biofuels from plant biomass [1], [2]. Although corn and sugarcane ethanol production in the US and Brazil have successfully established their markets, efforts to develop more sustainable (cellulosic; made from agricultural residues, perennial grasses, etc.) and more useful (drop-in; biodiesel, biobutanol, etc.) biofuels are still ongoing [3], [4], [5], [6], [7]. One of the challenges for producing next generation of biofuels is that microorganisms do not metabolize mixed sugars derived from cellulosic biomass as efficiently as glucose derived from corn and sugarcane. Corn stover hydrolysate, for example, consists of glucose (∼50%), xylose (∼40%), and some minor sugars (less than 10% of total sugars) such as fructose, arabinose, and galactose [8].

*Saccharomyces cerevisiae*, which is currently being used to produce corn ethanol, does not natively assimilate xylose [9]. Two xylose-assimilating pathways have been identified from other fungi or bacteria [10]. Introduction of the fungal pathway from *Scheffersomyces stipitis* or other fungi, consisting of xylose reductase (XR, coded by *XYL1*) and xylitol dehydrogenase (XDH, coded by *XYL2*), into *S. cerevisiae* is the most common strategy to engineer xylose-assimilating *S. cerevisiae* [11], [12], [13], [14], [15]. Xylose isomerase, mostly found in bacteria, is the other pathway that has also been functionally expressed in *S. cerevisiae* [16], [17], [18], [19], [20], [21]. Both pathways are often combined with the overexpression of xylulose kinase (*S. cerevisiae XKS1* or *Sch. stipitis XYL3*), which facilitates fluxes from the heterologous xylose-assimilating pathway to the native pentose phosphate pathway [14], [21]. The xylose fermentation by these engineered *S. cerevisiae* strains, however, was not as efficient as glucose fermentation in ethanol yield and productivity [11]. This limitation has prevented the engineered yeast from being employed for producing advanced biofuels including cellulosic ethanol.

Rational metabolic engineering strategies have been performed to improve the xylose metabolism of *S. cerevisiae* engineered by the heterologous xylose-assimilating pathway. First, overexpression of endogenous hexose transporters or heterologous xylose-specific transporters was shown to improve xylose consumption by the engineered *S. cerevisiae* [22], [23], [24]. Second, expression levels, codon usage, or cofactor preference of xylose-assimilating genes/enzymes from various microorganisms have been manipulated to build a better xylose-assimilating pathway [18], [25], [26], [27], [28]. Third, overexpression of single or multiple genes (*XKS1*, *TAL1*, etc.) in the pentose phosphate pathway resulted in improved xylose assimilation rate [14], [29]. Lastly, other metabolic pathways such as acetate biosynthesis and ammonia assimilation were studied to prevent acetate accumulation (*ALD6* deletion) [30] and to improve cofactor regenerations (*GDH2* overexpression) [31] during xylose metabolism, respectively.

Meanwhile, inverse metabolic engineering [32] strategies have been explored to deal with the unknown complexities of xylose metabolism that could not be captured by the rational approaches. Numerous studies developed strains with improved xylose utilization through laboratory evolution [33], [34], [35], [36]. Genome library screening, random transposon mutagenesis, and transcriptome analysis identified a number of candidate genes that could be deleted or overexpressed for improved xylose metabolism [37], [38], [39], [40], [41]. For example, overexpression of *XYL2*, *XKS1*, or *TAL1* was sufficient to improve xylose fermentation [38], [39], [41]. Some genes of unknown function, such as *PHO13* and *YLR042C*, were also found as deletion targets that improve xylose metabolism [38], [39].

In the present study, we integrated both rational and inverse metabolic engineering strategies to identify sufficient genetic perturbations facilitating rapid and efficient xylose metabolism by engineered *S. cerevisiae*. First, we optimized the xylose-assimilating pathway to maximize the ethanol yield without xylitol accumulation. Second, the strain was subjected to evolutionary engineering to overcome the xylose toxicity that was observed when the engineered *S. cerevisiae* strains were cultivated on above 10 g/l of xylose. Third, we resolved the acetate accumulation that interfered with xylose metabolism under high xylose concentrations. We demonstrated impressive xylose-fermenting capabilities of the final engineered strain under various sugar conditions.

## Results

### Xylose Reductase (XR) Activity Controls Xylose Consumption Rate of Engineered *S. cerevisiae*

**Figure 1:**
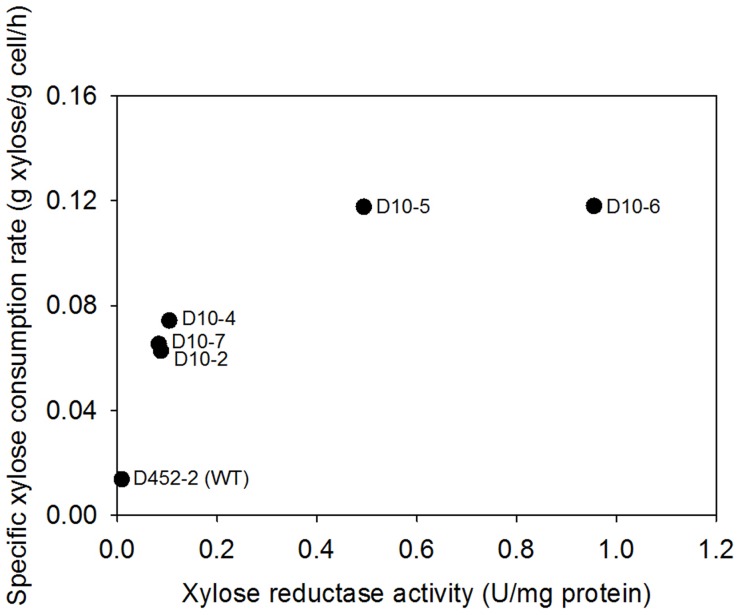
Effect of xylose reductase (XR) activity on the specific xylose consumption rates of various *S. cerevisiae* D452-2 strains (D10) expressing different copy numbers of *XYL1* derived from *Sch. stipitis*.

We hypothesized that xylose consumption rates by engineered *S. cerevisiae* might be determined by the first enzyme reaction catalyzed by XR (*XYL1*) when cofactors are sufficient for the reaction. To the hypothesis, we constructed various strains that exhibited different XR activities through random integration of the *XYL1* gene at the multiple Ty2 LTR sequences (δ elements) of the genome of *S. cerevisiae* D452-2. We selected 8 integrants (D10-1 to D10-8), and tested XR activities of crude cell extracts from the strains. While 6 transformants showed XR activity at 0.08–0.20 U/mg protein, 2 transformants had approximately 4 times (D10-5) and 7.5 times (D10-6) higher XR activity, suggesting that *XYL1* was integrated into multiple sites in the genome of the D10-5 and D10-6 strains. We selected 5 representative *XYL1* integrants and examined their xylose consumption rates in comparison to the wild type strain. The xylose consumption rates of the integrants were determined when they were growing aerobically on a mixture of glucose and xylose. After the cells converted all glucose into ethanol, the produced ethanol was simultaneously co-consumed with xylose. Because the integrants lacked a xylose metabolic pathway, the consumed xylose was secreted as xylitol at a constant rate, using the ethanol as the energy source [42], [43]. The integrants that exhibited high XR activities also had higher specific xylose consumption rates than the others (Figure 1) before the xylose consumption rate was saturated (D10-5 and D10-6). The wild type strain also had a basal level for specific xylose consumption rate by the action of endogenous aldose reductases. Quantitative PCR results suggested that the D10-6 strain that showed the highest XR activity had approximately 6 copies of *XYL1* in the genome.

### Construction of Various Xylose-assimilating Strains by Altering Copy Numbers of Xylose-assimilating Genes

Our previous study had suggested that high expression levels of *XYL2*, achieved by a strong promoter (*PGK1*), prevent xylitol accumulation and enhance ethanol production by engineered *S. cerevisiae* strains expressing *XYL1*, *XYL2*, and *XYL3* [41]. In this present study, we wanted to discover how relative expression levels of *XYL2* and *XYL1* would affect xylose fermentation phenotypes by engineered *S. cerevisiae*. Using the D10-6 strain that showed the highest XR activity through multiple integration of *XYL1* at δ sequences as a starting point, we then developed the SR6 strain by integrating an expression cassette containing *XYL1*, *XYL2*, and *XYL3* at the *URA3* locus. The SR6 strain, therefore, was used to represent a strain expressing *XYL1* at a higher level than *XYL2* and *XYL3*, resulting unbalanced high expression levels of *XYL1* as compared to *XYL2* and *XYL3*. In addition, the SR7 strain was constructed by integrating an additional copy of *XYL2* and *XYL3* into the SR6 strain. Unlike the SR6 strain, the SR7 strain represented a strain expressing the three genes at high and balanced levels.

**Figure 2:**
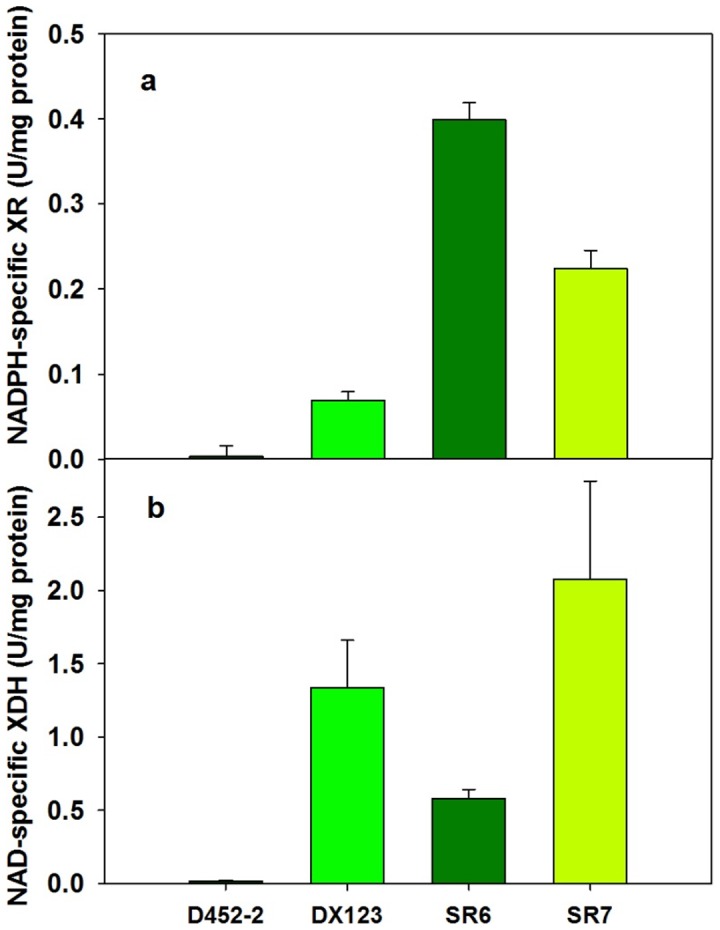
NADPH-specific xylose reductase (XR) activity and NAD-specific xylitol dehydrogenase (XDH) activity of three xylose-assimilating strains expressing different copy numbers of *XYL1, XYL2,* and *XYL3*. D452-2, wild type *S. cerevisiae*; DX123, D452-2 expressing one copy of *XYL1*, *XYL2*, and *XYL3*; SR6, D452-2 expressing multiple copies of *XYL1* and one copy of *XYL2* and *XYL3*; SR7, expressing multiple copies of *XYL1* and two copies of *XYL2* and *XYL3.* The figure illustrates the means and standard deviations of triplicate experiments.

Enzyme activities of XR and XDH of the SR6 and SR7 strains were compared to those of the DX123 strain [41] which had one integrated copy of *XYL1*, *XYL2*, and *XYL3*. Variations in copy numbers of *XYL1* and *XYL2* in the DX123, SR6, and SR7 strains resulted in altered enzymatic activities of XR and XDH (Figure 2). The SR6 strain had about 4 times higher XR activity than the DX123 strain, due to the multiple integrations of *XYL1* in the SR6 strain. The SR7 strain had approximately 2 times higher XDH activity than the DX123 strain, suggesting duplicate copies of *XYL2* in the SR7 strain, as we designed. Some results, however, did not correlate with the genomic copy numbers. The SR6 strain had lower XDH activity than the DX123 strain with the same copy numbers of *XYL2*, and the SR7 strain had lower XR activity than the SR6 strain with the same copy numbers of *XYL1*. We speculated that there might be an interaction between the XR and XDH activity when one is much higher than the other.

### Expression Levels of Xylose Reductase (XR) and Xylitol Dehydrogenase (XDH) Governed Xylose Fermentation Patterns by Engineered *S. cerevisiae*

**Figure 3:**
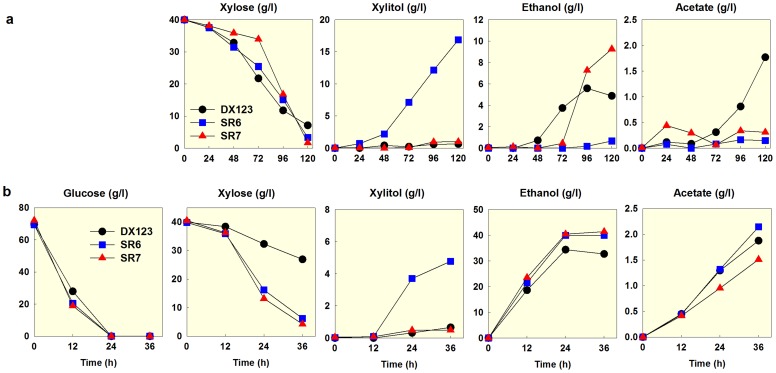
Fermentation profiles of three xylose-assimilating strains in YP media containing 40 g/l xylose (a) and a mixture of 70 g/l glucose and 40 g/l xylose (b). DX123, D452-2 expressing one copy of *XYL1*, *XYL2*, and *XYL3*; SR6, D452-2 expressing multiple copies of *XYL1* and one copy of *XYL2* and *XYL3*; SR7, expressing multiple copies of *XYL1* and two copies of *XYL2* and *XYL3*. All fermentations were performed under an oxygen-limited condition (100 rpm). An initial cell density was adjusted to 0.3 g/l. The figure illustrates the means of duplicate fermentations for each strain.

We investigated how the various xylose-fermenting strains with different copy numbers of *XYL1*, *XYL2*, and *XYL3* ferment xylose or a mixture of glucose and xylose under oxygen-limited conditions. When fermenting xylose as a sole carbon source, all three strains showed similar specific xylose consumption rates (0.16–0.21 g xylose/g cell/h; Figure 3a), possibly due to limitations in the cofactor regeneration for XR. Their fermentation profiles, however, were substantially different. The SR6 strain expressing high levels of *XYL1* accumulated large amounts of xylitol (0.36 g xylitol/g xylose) without producing ethanol. On the contrary, the DX123 and SR7 strains with balanced expressions of *XYL1* and *XYL2* produced ethanol and accumulated little amounts of xylitol (0.02–0.03 g xylitol/g xylose). The SR7 strain with a high and balanced expression of the xylose pathway produced the highest amount of ethanol with a yield of 0.24 g ethanol/g xylose. While the SR7 strain produced negligible amounts of acetate during xylose fermentation, the DX123 strain produced more than 1 g/l of acetate, suggesting that the acetate accumulation is associated with inefficient xylose fermentation. Higher acetate concentration could decrease the medium pH to its pKa value (4.8) when acetate becomes toxic to yeast [44].

The differences among the DX123, SR6, and SR7 strains became more obvious when fermenting a mixture of glucose (70 g/L) and xylose (40 g/L), representing general sugar compositions of cellulosic biomass (Figure 3b). While their glucose consumption rates were similar, the specific xylose consumption rate (0.11 g xylose/g cell/h) of the DX123 strain was much slower than those (0.22–0.25 g xylose/g cell/h) by the other strains, resulting in low ethanol production by the DX123 strain. The DX123 and SR7 strains with balanced expressions of *XYL1* and *XYL2* did not accumulate xylitol significantly (0.01–0.05 g/g xylose), as expected. Interestingly, the SR6 strain expressing higher *XYL1* than *XYL2* produced less xylitol (0.13 g/g xylose) than when fermenting only xylose (0.48 g/g xylose). As a result, the SR6 strain produced as much ethanol as the SR7 strain when fermenting a mixture of glucose and xylose. This improved xylose fermentation from a glucose and xylose mixture by the SR6 strain as well as the SR7 strain suggested that high expression levels of *XYL1* provide benefits in the xylose consumption rate when the required cofactors are regenerated efficiently through the glucose metabolism [45], [46].

### High Concentration of Xylose Inhibits its Metabolism by Engineered *S. cerevisiae*

**Figure 4:**
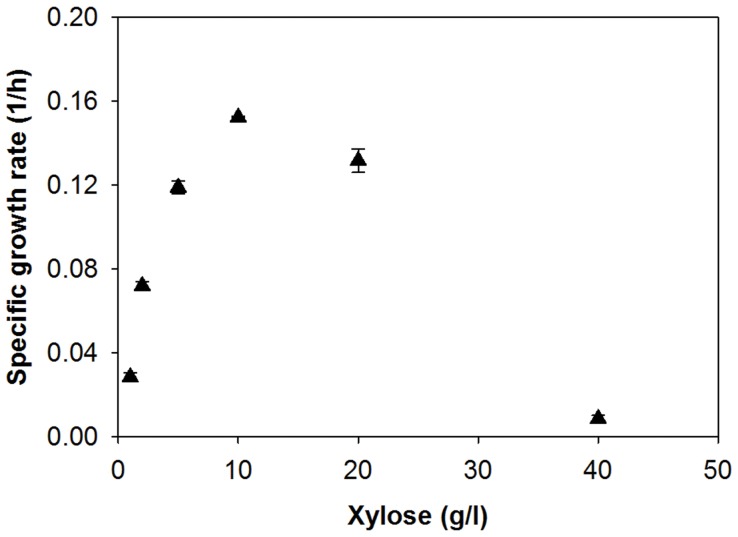
Effect of xylose concentration on the specific growth rates of engineered *S. cerevisiae* SR7 expressing a xylose assimilation pathway consisting of *XYL1, XYL2,* and *XYL3*. The figure illustrates the means and standard deviations of duplicate experiments.

When cultured in 40 g/l xylose as a sole carbon source (Figure 3a), the SR7 strain exhibited a long lag time, suggesting that some limitations or inhibitory mechanisms hindering xylose consumption might exist in the SR7 strain. We investigated if the delayed xylose consumption was related to initial xylose concentrations (Figure 4). When the initial xylose concentration was increased from 1 to 10 g/l, the specific growth rates increased as well. From 20 g/l xylose, however, the growth rate decreased drastically as the initial xylose concentration increased. When the SR7 strain was cultured in 40 g/l xylose, no significant cell growth was observed during 24 hours of incubation. This finding suggested that high concentrations of xylose severely inhibit its metabolism and the cell growth of engineered *S. cerevisiae* expressing *XYL1*, *XYL2*, and *XYL3*. The engineered strains may need to overcome the inhibition factors to start metabolizing 40 g/l xylose, resulting in a long lag time. It is uncertain if the inhibitory effects are completely resolved once the cells start metabolizing the xylose.

### Evolutionary Engineering of Xylose-fermenting *S. cerevisiae*

We hypothesized that growth inhibition by high concentrations of xylose would be a strong selective pressure for the isolation of suppressor mutants that have improved growth in xylose. To test this hypothesis, we carried out evolutionary engineering of the SR7 strain by inoculating in 40 g/l xylose at an initial optical density at 600 nm (OD_600_) of 0.1, and by transferring the culture to fresh media when the cells reached late log phase (OD_600_ of 5) to avoid the growth of non-desired mutants like ethanol consumers. The serial subcultures were repeated until changes in the phenotypes of each culture stabilized, and three independent sets of serial subcultures were performed.

**Figure 5:**
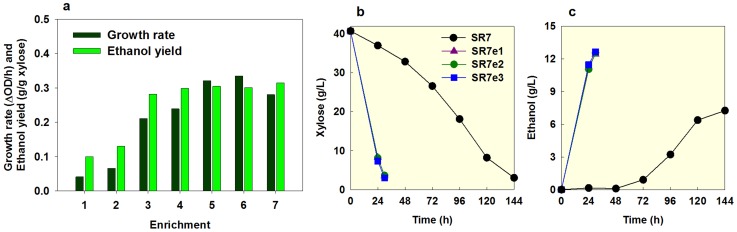
Evolution of the SR7 strain by serial subculturing on xylose. (a) Changes in the growth rate and ethanol yield during serial subcultures of engineered *S. cerevisiae* SR7 expressing a xylose assimilation pathway (*XYL1*, *XYL2*, and *XYL3*) in 40 g/l xylose (YPX40). (b and c) The xylose fermentation capability of three single colonies (SR7e1, SR7e2, and SR7e3), isolated from the last subculture, were evaluated in YPX40 as compared to the wild type SR7 (the means of duplicate experiments for each strain). All serial subcultures and fermentations were performed under an oxygen-limited condition (100 rpm). An initial cell density for serial subcultures or fermentations was adjusted to 0.03 g/l and 0.3 g/l, respectively.

Between the second and third subcultures of the SR7 strain, there was a huge improvement in growth rate and ethanol yield, suggesting that adaptation or advantageous mutations happened in the evolved strains (Figure 5a). Throughout the later subcultures, significant changes were not observed. Three single colony isolates (SR7e1, SR7e2, and SR7e3) were isolated from the last subcultures of each set. After pre-culturing in glucose to eliminate an adaptation effect, the three evolved strains were evaluated in the fermentation experiments with 40 g/l xylose as compared with the parental strain (SR7) (Figure 5b and c). All evolved strains exhibited significantly improved phenotypes that fermented xylose much more efficiently than the SR7 strain. As the fermentation time scale decreased from 5 days to approximately 1 day, the specific xylose consumption rate of the evolved strains improved from 0.19 to 0.66 (g xylose/g cell/h). Moreover, the ethanol yield increased drastically from 0.20 to 0.35 (g ethanol/g xylose) as well.

### Identification of Genetic Elements Associated with Improved Xylose Metabolism of the Evolved SR7 Strains

The whole genome of the wild type SR7 and the three evolved SR7 strains were sequenced and compared to discover genetic changes of the evolved strains which might be responsible for their improved phenotypes. First, we identified approximately 20 single nucleotide polymorphisms (SNPs) unique to the each evolved SR7 strain relative to the wild type SR7 strain (Table S1). A majority of the SNPs were intergenic (non-coding regions) or synonymous (silent substitutions). Among 5 genes containing non-synonymous SNPs in the evolved strains, only *PHO13*, the gene coding for alkaline phosphatase, was shared in all three evolved strains, as summarized in Table 1. In the same *PHO13* gene, the SR7e1 and SR7e2 had the same mutation (Gly166Arg), while the SR7e3 had a different mutation (Gly253Asp).

**Table 1:** SNPs identified in the three evolved SR7 strains as compared with the wild type SR7.

Non-synonymous SNPs in CDS	SR7e1	SR7e2	SR7e3
*PHO13*	Gly166Arg	Gly166Arg	Gly253Asp
*TUB2*	Ala110Ser	Ala110Ser	
*GLG1*			Phe364Leu
*YOL014W*		Phe6Leu	Phe6Leu
*DOG2*		Val147Leu	Val147Leu
Total SNPs in CDS	6	12	10
Intergenic SNPs	7	9	4
Other SNPs*	1	2	4
Total SNPs	14	23	18

* Telomeric repeats or a replication origin.

**Figure 6:**
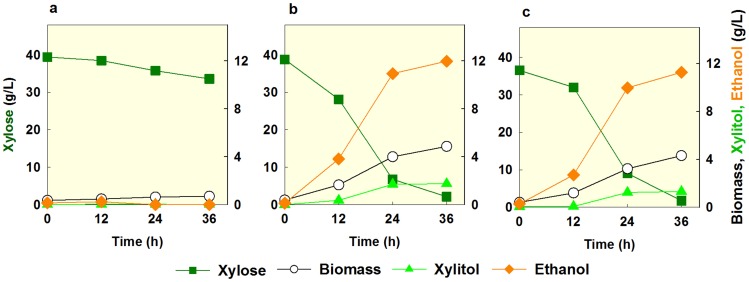
Comparison of wild type SR7 (a), SR7e3 (b), and SR7 *pho13Δ* (c) when fermenting 40 g/l xylose (YPX40) under an oxygen-limited condition. An initial cell density was adjusted to 0.3 g/l. The figure illustrates the means of duplicate experiments for each strain.

Although the biological function of the *PHO13* gene was not clearly elucidated, three previous studies reported that the deletion of *PHO13* could improve xylose fermentation by engineered *S. cerevisiae* [38], [47], [48]. Based on the previous findings, we hypothesized that the two different alleles of *PHO13* in the evolved strains might be loss of function mutations. To test this, we constructed SR7 *pho13Δ* and then compared the xylose fermentation profiles of the SR7, SR7e3, and SR7 *pho13Δ* strains using 40 g/l xylose (Figure 6). While the wild type SR7 strain did not show any significant metabolic activity during 36 h of incubation, both the SR7e3 and SR7 *pho13Δ* strains consumed most of the xylose and produced ethanol efficiently. The identical metabolic patterns of the SR7e3 and SR7 *pho13Δ* strains during xylose metabolism suggested that the improved phenotypes of the evolved strains might be caused by loss of the function of *PHO13*. Furthermore, other mutations besides *PHO13* might cause a marginal effect on xylose metabolism.

### Deletion of *PHO13* in Various Xylose-assimilating Strains Confirms Importance of the Pathway Optimization

**Figure 7:**
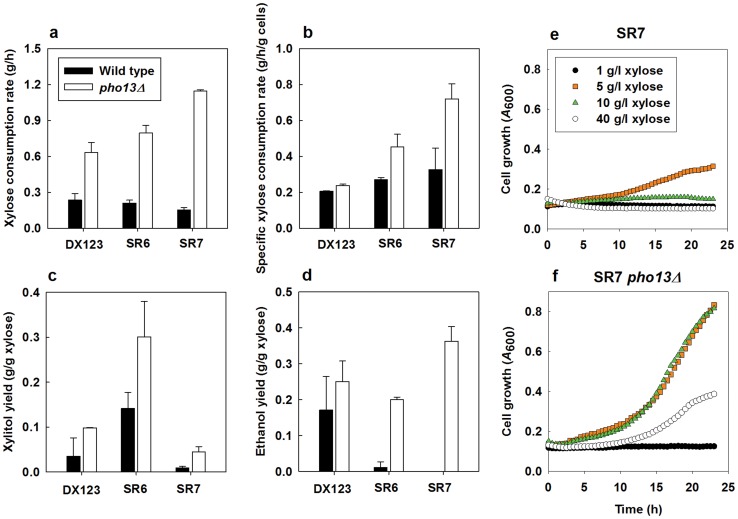
*PHO13* deletion affects xylose fermentation differently in various strain backgrounds. (a-d) Xylose fermentation parameters in three different xylose-assimilating strains and (e and f) growth patterns of the SR7 strain in various xylose concentrations: a, xylose consumption rate (g/h); b, specific xylose consumption rate (g/g cell/h); c, xylitol yield (g/g xylose); d, ethanol yield (g/g xylose); e, cell growth of SR7; f, cell growth of SR7 *pho13Δ*. All parameters were calculated at 36 h during 40 g/l xylose fermentation. An initial cell density was adjusted to 0.3 g/l (a-d) or 0.03 g/l (e-f). All experiments were duplicated.

We tested how the deletion of *PHO13* would affect xylose metabolism of the DX123, SR6, and SR7 strains, which express different xylose-assimilating pathways. All of the *pho13Δ* mutants of the DX123, SR6, and SR7 strains showed improved xylose consumption rates and ethanol yields when fermenting 40 g/l of xylose (Figure 7). However, the degree of the improvement and xylitol yields varied among strains depending on their xylose-assimilating pathways. Specifically, the SR6 strain, which accumulates a large amount of xylitol due to relatively low expression level of *XYL2* compared to *XYL1*, produced twice the amount of xylitol after deleting *PHO13*. During 40 g/l xylose fermentation for 36 h, the SR7 strain with balanced and high expressions of xylose-assimilating pathway showed the highest improvement after the deletion of *PHO13* among the three strains as shown in Figure 7. The SR7 *pho13Δ* strain yielded the highest xylose-assimilating rate and the highest ethanol yield while producing the least amount of xylitol. These results suggest that the deletion of *PHO13* alleviates limitations in xylose consumption by *S. cerevisiae* expressing the heterologous xylose-assimilating pathway (*XYL1*, *XYL2*, and *XYL3*). However, it did not alter the original fermentation patterns determined by the expression levels of *XYL1*, *XYL2*, and *XYL3*.

The inhibitory action of Pho13p on xylose fermentation might be related to the xylose toxicity (the delayed cell growth in high concentrations of xylose). After deleting *PHO13* in the SR7 strain, the lag time in 40 g/l of xylose decreased from >24 h to 10 h (Figure 7f and e). However, the growth rate of the SR7 *pho13Δ* mutant in 40 g/l of xylose was still lower than the rates in 5 or 10 g/l of xylose (Figure 7 e). Because the xylose toxicity was not completely resolved by the deletion of *PHO13*, there might be other enzymes or mechanisms causing the xylose toxicity than the Pho13p activity.

### Mechanism of the Inhibitory Action of Pho13p on Xylose Metabolism

**Figure 8:**
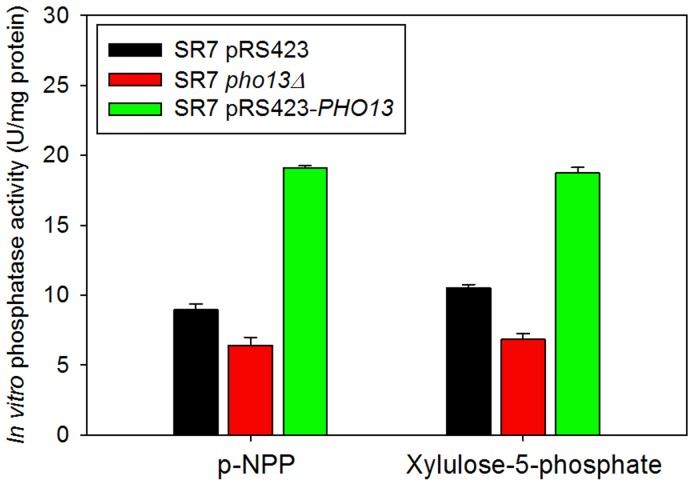
*In vitro* phosphatase activity of crude cell extracts from SR7 control (pRS423), SR7 *pho13Δ*, and *PHO13*-overexpressing SR7 (SR7 pRS423-*PHO13*) on p-nitrophenyl phosphate (p-NPP, an artificial phosphatase substrate, a positive control) and xylulose-5-phosphate. The figure illustrates the means and standard deviations of triplicate experiments.

Although the advantage of the *PHO13* deletion for xylose metabolism has been reported in previous studies [38], [47], [48], the mechanism has not been clearly described. A prior study characterized *S. cerevisiae* Pho13p as a member of the haloacid dehalogenase (HAD)-like hydrolase superfamily, and proposed phosphatase activity of Pho13p on xylulose-5-phosphate [49] as the inhibitory mechanism of Pho13p on xylose metabolism. To test this hypothesis, we measured *in vitro* phosphatase activity of crude enzyme extracts from the SR7 pRS423 (control), SR7 *pho13Δ*, and SR7 pRS423-*PHO13* (*PHO13*-overexpressing mutant) strains using xylulose-5-phosphate as a substrate. *para*-nitrophenyl phosphate (p-NPP), an artificial phosphatase substrate, was also used as a positive control [50]. Phosphatase activities of the enzyme extracts on both p-NPP and xylulose-5-phosphate showed a similar pattern (Figure 8): deletion of *PHO13* had a mild effect, whereas overexpression of *PHO13* increased phosphatase activity about 2-fold. The phosphatase activities shown in the SR7 *pho13Δ* mutant was speculated because of numerous phosphatase enzymes other than Pho13p. These results confirm that Pho13p has phosphatase activity on xylulose-5-phosphate *in vitro*, and suggest that *in vivo* Pho13p, together with overexpressed XK, might forms a futile cycle that leads to depletion of ATP and subsequent inhibition of xylose metabolism.

### Disruption of Acetaldehyde Dehydrogenase (*ALD6*) Facilitates Efficient Xylose Fermentation by Preventing Acetate Accumulation

**Figure 9:**
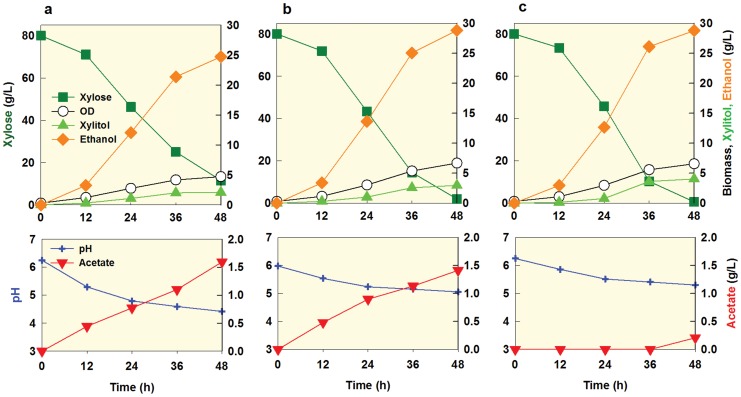
Fermentation profiles and pH changes of SR7e3 (a), SR7e3 with pH control (b), and SR7e3 *ald6Δ* (i.e. SR8, c) when fermenting 80 g/l xylose (YPX80) under an oxygen-limited condition. An initial cell density was adjusted to 0.3 g/l.

During dilute acid pretreatment of cellulosic biomass, hemicellulose (mostly xylose) is more efficiently hydrolysed than cellulose. As a result, a liquid fraction of corn stover hydrolysates, for example, contains high xylose (∼80 g/l) and low glucose (∼20 g/l) [51]. Although the evolved strain SR7e3 exhibited very efficient xylose-fermenting capability at 40 g/l xylose, delayed xylose consumption was observed in the later part of fermentation when higher xylose concentrations were used, such as 80 g/l xylose (Figure 9a). During fermenting 80 g/l of xylose, the SR7e3 strain accumulated acetate, resulting in a gradual decrease of medium pH from 6.1 to 4.9 during 24 hours. Considering that the pKa value of acetic acid is 4.8, the pH drop of the medium might have increased the undissociated (protonated) form of acetic acid, which can freely diffuse across cell membranes and cause cellular toxicity. In a buffered medium at pH 6.0, however, the xylose fermentation by SR7e3 was successfully completed while accumulating the same amount of acetic acid as before (Figure 9b), suggesting that acetate toxicity was inhibiting xylose metabolism in the unbuffered medium.

As an alternative solution, acetic acid accumulation can be prevented by a genetic perturbation. Because disruption of *ALD6*, a major gene coding for acetaldehyde dehydrogenase, was shown to reduce acetate production previously [30], [52], [53], [54], [55], we disrupted *ALD6* in the SR7e3 strain. The resulting strain (SR8) indeed did not produce acetic acid when fermenting 80 g/l of xylose and the medium pH was maintained above 5 throughout the fermentation (Figure 9c). As a result, the SR8 strain was able to ferment xylose as efficiently as when using a buffered medium by the strain without *ALD6* disruption (SR7e3) (Figure 9b). Unlike a previous study, we did not observe any changes in the lag time of the *ALD6* knockout strain when grown in xylose as a sole carbon source [56].

We tested the advantage of the *ALD6* knockout by comparing fermentation profiles by the SR7e3 and SR8 (SR7e3 *ald6Δ*) strains under various sugar conditions (Table 2). While acetate accumulation by the SR7e3 strain varied under different conditions, the SR8 strain did not accumulate acetate under all conditions tested in this study. Other than the acetate accumulation, any significant difference between the SR7e3 and SR8 strains was not observed when fermenting 40 g/l xylose. However, in all higher sugar concentrations, the SR8 strain showed higher growth rates, volumetric xylose consumption rates, and volumetric ethanol productivities than the SR7e3 strain. Moreover, during fermentation of 20 g/l glucose and 80 g/l xylose, the SR8 strain yielded an 8% higher specific xylose consumption rate (0.83 g xylose/g cell/h) than the SR7e3 strain (0.77 g xylose/g cell/h). The elimination of acetic acid accumulation by the *ALD6* knockout did not change the ethanol yield but slightly increased the biomass, xylitol, and glycerol yields, regardless of the sugar conditions.

**Table 2:** Fermentation profiles of SR7e3 and SR8 (SR7e3 *ald6Δ*) in various sugar conditions^1)^.

	40 g/l xylose (at 24 h)		80 g/l xylose (at 36 h)		70 g/l glucose and 40 g/l xylose (at 24 h)		20 g/l glucose and 80 g/l xylose (at 30 h)	
	SR7e3	SR8	SR7e3	SR8	SR7e3	SR8	SR7e3	SR8
Acetate (g/l)	0.38±0.07	0.00±0.00	0.99±0.08	0.00±0.00	1.17±0.03	0.00±0.00	1.36±0.15	0.00±0.00
pH			4.60±0.01	5.41±0.01			4.69±0.13	5.74±0.01
*µ*	0.10±0.00	0.09±0.00	0.08±0.02	0.09±0.01	0.09±0.01	0.11±0.00	0.08±0.01	0.11±0.01
*r*_Xylose_	1.34±0.07	1.30±0.02	1.75±0.12	2.10±0.03	1.20±0.01	1.68±0.00	1.69±0.03	2.54±0.11
*r*_Xylose_*	0.78±0.16	0.87±0.20	0.74±0.03	0.74±0.06	0.43±0.03	0.60±0.00	0.77±0.02	0.83±0.02
*Y*_Ethanol_	0.30±0.06	0.31±0.05	0.37±0.03	0.37±0.00	0.42±0.01	0.41±0.00	0.39±0.01	0.39±0.01
*P*_Ethanol_	0.43±0.14	0.44±0.13	0.65±0.10	0.79±0.01	1.70±0.02	1.87±0.01	0.92±0.03	1.25±0.02
*P*_Ethanol_*	0.24±0.02	0.28±0.01	0.27±0.04	0.28±0.02	0.61±0.04	0.67±0.01	0.42±0.01	0.41±0.02

1 Fermentations were performed under oxygen-limited conditions (100 rpm) in YP medium (pH 6.6), and an initial cell density was 0.3 g/l (OD_600_ 1). All parameters were calculated when more than 90% of xylose was consumed at each condition.

Parameters: *µ*, specific growth rate (1/h); *r*_Xylose_, xylose consumption rate (g/l/h); *r*_Xylose_*, specific xylose consumption rate (g/g cell/h); *Y*_Ethanol_, ethanol yield (g/g sugars); *P*_Ethanol_, volumetric ethanol productivity (g/l/h); *P*_Ethanol_*, specific ethanol productivity (g/g cell/h).

## Discussion

Numerous hypotheses explaining slow and inefficient xylose assimilation by engineered *S. cerevisiae* expressing a xylose-assimilating pathway have been proposed. The lack of xylose-specific transporters [57] in *S. cerevisiae* is one of them. Xylose can be transported non-specifically by hexose transporters such as *GAL2* and *HXT7* [24], [58], [59], [60]. Because the expression of those transporters is repressed by glucose, xylose transport is completely inhibited by the presence of glucose [11]. Moreover, the non-specific transport might be a reason why xylose consumption rates reduce at the end of fermentation when xylose concentration is low [11], [61]. Based on our results, optimal aeration or glucose consumption improves xylose consumption rates of engineered *S. cerevisiae* strains expressing high levels of XR (Figure 1 and Figure 3b). This suggests that the ATP production and/or cofactor regeneration is limiting the rate of xylose metabolism determined by the activity of XR. Therefore, xylose transport might not be a limiting factor up to the rates of xylose consumption showed in this study.

As the second enzyme of the xylose-assimilating pathway, XDH (*XYL2*), requires NAD^+^ exclusively, the unbalanced cofactor requirement between XR and XDH has received considerable attention as a major limiting factor of xylose fermentation by engineered *S. cerevisiae* [62]. It has been hypothesized that the cofactor imbalance might result in xylitol accumulation and slow xylose assimilation [62]. Thus, many studies attempted to alter the cofactor specificity of either XR or XDH enzyme to balance their cofactor requirements [25], [26], [27], [30], [63], [64], [65], [66]. Other studies also tried to develop a pathway that helps the regeneration of the cofactors by overexpressing NADP^+^-dependent glyceraldehyde-3-phosphate dehydrogenase [67], NADH-dependent glutamate dehydrogenase [31], or water-forming NADH oxidase [68]. Although these studies were successful in deceasing xylitol yields, the improvements were not directly correlated with ethanol yields [62]. This suggests that the cofactor imbalance might not be the major cause of inefficient xylose fermentation.

Contrary to the redox imbalance theory, our results indicates that xylose fermentation profiles can be changed through modulating the expression levels of *XYL1*, *XYL2*, and *XYL3*, even without changing their cofactor preference. If the expression level of *XYL2* is high relative to *XYL1*, xylitol accumulation can be minimized. This conclusion is also consistent with our previous finding that high expression levels of *XYL2* reduce xylitol accumulation [41]. In the meantime, high expression levels of *XYL1* improve xylose consumption rates. The reduced xylitol yield and faster xylose assimilation by the high and balanced expression levels of *XYL1* and *XYL2*, therefore, enable a higher ethanol production by allowing more flux to the pathway and by generating more ATP. Moreover, only when enough ATP is generated by optimized expression levels of *XYL1* and *XYL2*, overexpression of the XK genes (*XYL3* or *XKS1*) might accelerate xylose assimilation rate without depleting intracellular ATP. The shortage of ATP was previously proposed as the reason why strong expressions of the XK genes were inhibitory to the growth of xylose-fermenting *S. cerevisiae* [38], [69], [70].

**Figure 10:**
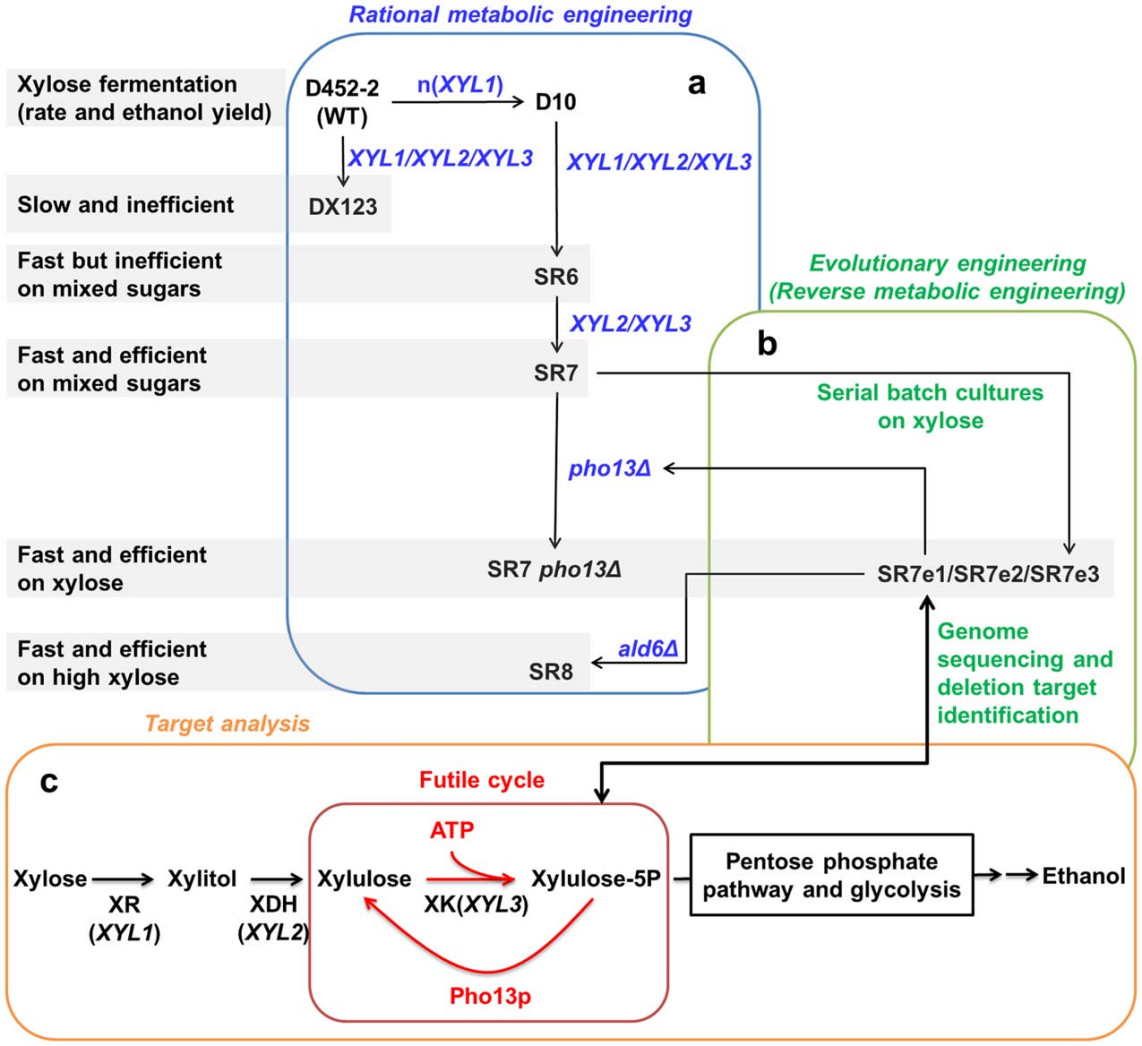
Summary of metabolic engineering strategies used in this study to develop efficient xylose-fermenting *S. cerevisiae.* (a) Rational metabolic engineering strategies to optimize a xylose-assimilating pathway consisting of the *XYL1*, *XYL2*, and *XYL3* genes and to overcome acetate toxicity (*ald6Δ*), (b) evolutionary engineering to isolate mutants (SR7e1, SR7e2, SR7e3) that grow faster on xylose and to identify genetic changes (*pho13Δ*) of the mutants through genome sequencing, and (c) targeted analysis to confirm inhibitory effect of Pho13p on xylose metabolism by engineered *S. cerevisiae*.

In addition to the optimized expression levels of *XYL1*, *XYL2* and *XYL3*, the deletion of the *PHO13* gene (*pho13*Δ) plays an important role in improving xylose consumption rates and ethanol yields. When paired with strong activity of the XK enzyme, the Pho13p activity may generate a futile cycle by dephosphorylating xylulose-5-phosphate, resulting in accelerated ATP depletion and flux blockage (Figure 10c). Although *pho13*Δ increases xylose consumption rates and ethanol yields of engineered *S. cerevisiae* strains regardless of the xylose-assimilating pathway, the improvement is most significant when the expression levels of *XYL1*, *XYL2*, and *XYL3* are optimized like the SR7 strain. This indicates that the advantageous effects of *pho13*Δ on xylose fermentation are strongly associated with the efficiency of the xylose-assimilating pathway. With the optimized xylose-assimilating pathway and the deletion of the *PHO13* gene, the SR7 *pho13Δ* strain fermented xylose very efficiently at higher specific ethanol productivity (0.25 g ethanol/g cell/h; Figure 6c) than any other rationally engineered *S. cerevisiae* strains reported previously (0.11–0.20 g ethanol/g cell/h) [48], [71], [72], [73]. Although there are three prior studies that reported very high specific ethanol productivities (0.32–0.77 g ethanol/g cell/h) from xylose by evolved strains of *S. cerevisiae* [36], [74], [75], their genetic changes which are responsible for the improved xylose metabolism are unknown.

Evolutionary engineering (or adaptive evolution) [76] is used to enrich desired phenotypes from spontaneously induced mutants or chemically mutagenized population by repeated batch cultures [77], [78] or extended continuous fermentations [36], [74] under selective conditions. Combined with genome sequencing, evolutionary engineering also provides a powerful tool for identifying unknown gene targets [32]. A recent study successfully identified three new genes regulating galactose metabolism through genome sequencing of evolved *S. cerevisiae* strains growing better on galactose [79]. In the present study, the *PHO13* gene was identified as a critical deletion target to improve xylose-fermenting capabilities of engineered *S. cerevisiae* as efficiently as the three evolved strains described above (Figure 10). These successful examples indicate that evolutionary engineering followed by genome sequencing can be used to obtain other industrially important phenotypes of interest for producing cellulosic fuels and chemicals.

### Conclusions

Optimization of the expression levels of *XYL1*, *XYL2*, and *XYL3*, and disruption of *PHO13* and *ALD6* enabled efficient xylose fermentation, which allowed co-fermentation of glucose and xylose at various concentrations. This simple set of genetic perturbations for developing an efficient xylose-fermenting laboratory *S. cerevisiae* strain could be used to engineer industrial *S. cerevisiae* strains for the production of cellulosic fuels and chemicals. New findings related to xylose toxicity and the xylulose-5-phosphate phosphatase activity of Pho13p will advance our understanding of xylose metabolism of engineered *S. cerevisiae* expressing a heterologous xylose-assimilating pathway. Lastly, this study provides the first example of a successful application of evolutionary engineering followed by genome sequencing to identify genetic elements that improve xylose-fermenting capabilities of engineered *S. cerevisiae*.

## Methods

### Media, Strains, and Plasmids

Cells were cultured in YP medium (10 g/l yeast extract and 20 g/l peptone) containing glucose (YPD), xylose (YPX), or a mixture of glucose and xylose (YPDX). The concentrations of the sugars were displayed as numbers following their initials (e.g., YPX40, YP medium containing 40 g/l xylose; YPD70D40, YP medium containing 70 g/l glucose and 40 g/l xylose), except YPD, which contained 20 g/l glucose.

All plasmids and strains used in this study are summarized in Table 3. D10 refers to the D10-1, D10-2, D10-3, D10-4, D10-5, and D10-6 strains generated by integrating *Hpa*I-treated pYS10 at the *leu2* locus of the D452-2 genome [80]. Due to Ty2 sequences adjacent to the *LEU2* gene in the pYS10 plasmid [81], some integrants (D10-5 and D10-6) which showed higher XR activity had multiple integrations of the plasmid at the Ty2 loci. Three xylose-fermenting *S. cerevisiae* strains (DX123, SR6, and SR7) expressing different copy numbers of xylose-metabolic genes were made as follows: the pSR6-X123 plasmid [41] containing the *XYL1*, *XYL2*, and *XYL3* genes was integrated at the *URA3* locus of the D452-2 and D10-6 strains, yielding the DX123 and SR6 strains, respectively. The pSR3-X23 plasmid containing the *XYL2* and *XYL3* genes was additionally integrated at the *HIS3* locus of the SR6 strain, yielding the SR7 strain. The SR7 strain was enriched during serial subcultures on YPX40, and three colonies (SR7e1, SR7e2, and SR7e3) from each evolved culture were isolated. To construct the *PHO13* deletion mutants, the *pho13Δ*::*KanMX4* cassette was PCR-amplified from the genomic DNA of the BY4742 *pho13Δ* strain (clone ID: 13933) of the Yeast Knockout Collection (Open Biosystems). The PCR products were purified and integrated to the DX123, SR6, and SR7 strains, and the resulting deletion mutants were selected on YPD containing 300 µg/ml of G418. To test *in-vitro* enzymatic activity of Pho13p, the SR7 strain was transformed by a multicopy plasmid (pRS423-*PHO13*) expressing the *PHO13* gene using its native promoter. To disrupt the *ALD6* gene, the pAUR_d_ALD6 plasmid [30] containing the truncated *ALD6* gene was integrated to the SR7e3 strain, and the resulting transformant (SR8) was selected on YPD containing 0.5 µg/ml of Aureobasidin A.

**Table 3:** Plasmids and strains used in this study.

	Characteristics	References
Plasmids		
pYS10	pRS305 *TDH3*_P_−*XYL1*−*TDH3*_T_	[83]
pSR3-X23	pRS403 *PGK1*_P_−*XYL2*−*PGK1*_T_ *TDH3*_P_−*XYL3*−*TDH3*_T_	This study
pSR6-X123	pRS306 *TDH3*_P_−*XYL1*−*TDH3*_T_ *PGK1*_P_−*XYL2*−*PGK1*_T_ *TDH3*_P_−*XYL3*−*TDH3*_T_	[41]
pRS423-*PHO13*	pRS423 containing the *PHO13* gene with its native promoter and terminator	This study
pAUR_d_ALD6	pAUR101 containing the truncated *ALD6* gene	[30]
*S. cerevisiae*		
D452-2	*MAT*α *leu2 ura3 his3*	[84]
D10	D452-2 *leu2*::*LEU2* pYS10	This study
DX123	D452-2 *ura3*::*URA3* pSR6-X123	[41]
SR6	D10-6 *ura3*::*URA3* pSR6-X123	This study
SR7	SR6 *his1*::*HIS1* pSR3-X23	This study
SR7e	Evolved strains of SR7 (SR7e1, SR7e2, and SR7e3)	This study
DX123 *pho13Δ*	DX123 *pho13Δ*::*KanMX4*	This study
SR6 *pho13Δ*	SR6 *pho13Δ*::*KanMX4*	This study
SR7 *pho13Δ*	SR7 *pho13Δ*::*KanMX4*	This study
SR7 control	SR7 pRS423	This study
SR7 pRS423-*PHO13*		This study
SR8	SR7e3 *ald6*::*AUR1-C* pAUR_d_ALD6	This study

### Quantitative PCR for Determining Genomic Copy Numbers of *XYL1* in the D10-6 Strain

The genomic DNA of the D10-6 strain was prepared with the YeaStar Genomic DNA Kit (Zymo Research, Orange, CA) and quantified by NanoDrop ND-1000 (Thermo Fisher Scientific, Wilmington, DE). Primers (5′-CTTGGACTTGTTGAGAGGTG-3′ and 5′-AACGAAGAGTAAGCGGTGAC-3′) for the detection of the *XYL1* gene were designed. Real-time PCR was performed in 96-well plates on a Lightcycler 480 instrument (Roche Applied Science, Indianapolis, IN) using SYBR Green I Master (Roche) as the manufacturer’s protocol. The concentration of the *XYL1* gene in the samples was quantified by a standard curve generated by the *XYL1* gene fragments (0.01, 0.1, 1, and 10 pg/µl) purified from the pYS10 plasmid treated with *Hind*III. Genomic copy numbers (x) of the *XYL1* gene in the genomic DNA samples were estimated by the following equation where a is the size of the *XYL1* gene fragment (1.03 kb), b is the size of the whole genome of *S. cerevisiae* (12000 kb), c is the concentration of the *XYL1* gene calculated by quantitative PCR (ng/µl), and d is the concentration of the genomic DNA samples (ng/µl): ax/b = c/d.

### Enzyme Assay

XR and XDH activities were determined as follows:

Cells were grown aerobically in YPD and harvested in exponential phase. The cells were washed with water, resuspended in Y-PER soultion (Pierce, Rockford, IL, USA), and incubated for 20 min at room temperature. After centrifugation of the cell suspension for 10 min at 4^o^C, the supernatant (crude enzyme extracts) was obtained and kept on ice. XR activity was tested in 1 ml of reaction solution containing 0.7 ml of 50 mM sodium phosphate buffer (pH 6.5), 0.1 ml of the cell extracts, 0.1 ml of 2 mM NADPH, and 0.1 ml of 1 M xylose. XDH activity was tested in 1 ml of reaction solution containing 0.7 ml of 50 mM Tris buffer (pH 9), 0.1 ml of the cell extracts, 0.1 ml of 2 mM NAD, and 0.1 ml of 1 M xylitol. The rate of absorbance change (Δabs/min) of the reaction solution was monitored at 340 nm by a spectrophotometer (Genesis 10; ThermoFisher Scientific, Waltham, MA). The conversion factors of NADPH and NADH were 0.2410 and 0.2227 µmol/Δabs, respectively (based on 1 ml reaction volume). One unit (1 U) of enzyme was defined by the conversion of 1 µmol NADPH or NAD per minute. The units were normalized by the amount of total protein (mg) measured by a BCA protein assay kit (Pierce).

Phosphatase activity was measured as described previously [82]. Cells were grown aerobically in YPD containing 200 ug/ml G418, and their crude cell extracts were prepared as mentioned above. Fifty-microliters of reaction solution contained 50 mM Tris-HCl (pH 7.5), 5 mM MgCl_2_, 1 mM DTT, 5 ul of the cell extracts, and 4 mM of a substrate (*para*-nitrophenylphosphate or xylulose-5-phosphate). The enzyme solution was incubated at 30°C for 30 min, and quenched by adding 250 ml of 2 mM EDTA. Inorganic phosphate released from the substrates was measured by a Malachite green phosphate assay kit (BioAssay Systems, Hayward, CA). One unit (1 U) of enzyme was defined by the liberation of 1 µmol phosphate per minute. The units were normalized by the amount of total protein (mg) used in the reaction solution.

### Specific Xylose Consumption Rates of the D10 Strains

The D10 strains harboring multiple genomic copies of *XYL1* were precultured in 5 ml YPD, and inoculated in 50 ml YPD40X40 at an initial cell density of 0.1 g/l. The cultures were incubated at 30°C and 250 rpm for 4 days for the cells to convert the xylose to xylitol while aerobically assimilating the ethanol produced from the glucose. Their specific xylose consumption rates (g/g cell/h) were calculated and are displayed in Figure 1.

### Specific Growth Rates of SR7 in Various Xylose Concentrations

The growth of SR7 was monitored by the Bioscreen C platereader system (Growth Curves USA, Piscataway, NJ). A YPD culture of SR7 was used to inoculate 250 ul of YP medium containing various concentrations of xylose in a honeycomb microplate at a cell density of 1 × 10^6^ cells/ml. The plate was incubated at 30°C and subjected to medium shaking for 24 h. The OD_600_ was monitored every 30 min, and the results were used to calculate the specific growth rates (1/h) during exponential phase (Figure 4).

### Fermentation

All fermentation experiments were performed by resuspending YPD-grown cells in 50 ml of fermentation media in a 250-ml erlenmeyer flask at an initial cell density of 0.3 g/l. The fermentation flasks were incubated at 30°C and 100 rpm to maintain an oxygen-limited condition, which allows the highest ethanol productivity from xylose with a decent ethanol yield. Every 12 hours of fermentation, 1 ml of the samples was taken to measure the OD_600_, pH, and metabolite concentrations.

### HPLC Analysis

The glucose, xylose, xylitol, glycerol, acetate and ethanol concentrations of the samples were analyzed by high performance liquid chromatography (HPLC, Agilent Technologies 1200 Series) equipped with a refractive index detector and a Rezex ROA-Organic Acid H+ (8%) column (Phenomenex Inc., Torrance, CA). The column was eluted with 0.005 N of H_2_SO_4_ at a flow rate of 0.6 ml/min at 50^o^C.

### Genome Sequencing and SNP Discovery

The genomic DNA of SR7, SR7e1, SR7e2, and SR7e3 was prepared with the YeaStar Genomic DNA Kit (Zymo Research), and its quality was confirmed on a 1% agarose gel. Barcoded library construction and genome sequencing using an Illumina HiSeq2000 machine were performed at the W. M. Keck Center for Comparative and Functional Genomics at the University of Illinois at Urbana-Champaign. The barcoded shotgun genomic DNA libraries were constructed with the TruSeq Sample Prep Kit following the manufacturer’s manual (Illumina, San Diego, CA), and then quantitated by fluorometry (Qubit), a bioanalyzer (Agilent), and quantitative PCR. The diluted libraries (10 mM) were pooled in equimolar concentration and multiplexed on a lane and sequenced from one end (single-reads) for 100 nt with an Illumina HiSeq2000 using SBS chemistry version 2. The raw data were processed with Casava 1.7.

Each sample yielded approximately 10 million reads, and the average quality scores per base were higher than 30 (Solexa scale: 40 =  highest, −15 =  lowest). All FASTQ files are deposited in the SRA. SNP analysis was performed using CLC Genomics Workbench version 5.1. Reads were trimmed based on quality scores using default program settings. The trimmed reads were mapped to an S288C reference sequence (obtained from Genbank). SR7, SR7e1, SR7e2, SR7e3 SNPs were identified using a coverage cutoff of 10, 90% minimum variant frequency, expected number of variants 1. Each strain yielded approximately 5,000 SNPs relative to S288C. SNPs unique to SR7e1, SR7e2, SR7e3 relative to SR7 were then identified by comparing these SNP files, as listed in Table S1.

## Supporting Information

Table S1 **SNPs unique to SR7e1, SR7e2, SR7e3 relative to SR7.**
(XLSX)
